# Bisulfite Conversion of DNA: Performance Comparison of Different Kits and Methylation Quantitation of Epigenetic Biomarkers that Have the Potential to Be Used in Non-Invasive Prenatal Testing

**DOI:** 10.1371/journal.pone.0135058

**Published:** 2015-08-06

**Authors:** Chrysanthia A. Leontiou, Michael D. Hadjidaniel, Petros Mina, Pavlos Antoniou, Marios Ioannides, Philippos C. Patsalis

**Affiliations:** 1 Translational Genetics Team, The Cyprus Institute of Neurology & Genetics, Nicosia, Cyprus; 2 NIPD Genetics Ltd, Nicosia, Cyprus; 3 The Ministry of Health, Nicosia, Cyprus; University of Bonn, Institut of experimental hematology and transfusion medicine, GERMANY

## Abstract

**Introduction:**

Epigenetic alterations, including DNA methylation, play an important role in the regulation of gene expression. Several methods exist for evaluating DNA methylation, but bisulfite sequencing remains the gold standard by which base-pair resolution of CpG methylation is achieved. The challenge of the method is that the desired outcome (conversion of unmethylated cytosines) positively correlates with the undesired side effects (DNA degradation and inappropriate conversion), thus several commercial kits try to adjust a balance between the two. The aim of this study was to compare the performance of four bisulfite conversion kits [Premium Bisulfite kit (Diagenode), EpiTect Bisulfite kit (Qiagen), MethylEdge Bisulfite Conversion System (Promega) and BisulFlash DNA Modification kit (Epigentek)] regarding conversion efficiency, DNA degradation and conversion specificity.

**Methods:**

Performance was tested by combining fully methylated and fully unmethylated λ-DNA controls in a series of spikes by means of Sanger sequencing (0%, 25%, 50% and 100% methylated spikes) and Next-Generation Sequencing (0%, 3%, 5%, 7%, 10%, 25%, 50% and 100% methylated spikes). We also studied the methylation status of two of our previously published differentially methylated regions (DMRs) at base resolution by using spikes of chorionic villus sample in whole blood.

**Results:**

The kits studied showed different but comparable results regarding DNA degradation, conversion efficiency and conversion specificity. However, the best performance was observed with the MethylEdge Bisulfite Conversion System (Promega) followed by the Premium Bisulfite kit (Diagenode). The DMRs, EP6 and EP10, were confirmed to be hypermethylated in the CVS and hypomethylated in whole blood.

**Conclusion:**

Our findings indicate that the MethylEdge Bisulfite Conversion System (Promega) was shown to have the best performance among the kits. In addition, the methylation level of two of our DMRs, EP6 and EP10, was confirmed. Finally, we showed that bisulfite amplicon sequencing is a suitable approach for methylation analysis of targeted regions.

## Introduction

Prenatal testing is a significant part of modern obstetric care and its primary aim is the diagnosis of fetal genetic abnormalities [1]. In Europe, the prevalence of chromosomal abnormalities for all pregnancies is 3.6 per 1,000 births [2]. Trisomies 21, 18 and 13 and sex chromosome anomalies are the most common identified among the live births with aneuploidies [3]. Currently, the main goal of prenatal testing is to provide parents with the choice to abort a fetus with the diagnosed condition or to prepare psychologically, socially, financially and medically for a child with a health problem or disability, or for the likelihood of a stillbirth [4]. The most common methods used for prenatal diagnosis are chorionic villi sampling (CVS) during the first trimester and amniocentesis during the second trimester, with their diagnostic accuracy estimated to be 98 to 99% [5, 6]. Both of these procedures are invasive with a significant risk of fetal loss, between 0.5 to 1% of all tested cases [7–9]. Therefore, these invasive tests are currently performed only in high-risk pregnancies or in pregnancies with increased maternal age and/or family history of having a child with an inherited disease.

The discovery of free fetal DNA in maternal circulation in 1997, marked a significant step towards the development of non-invasive prenatal diagnostic assays [10]. Compared to fetal cells, cell-free fetal DNA (cffDNA) is relatively more abundant in the maternal plasma and thus it represented a promising molecular tool for non-invasive prenatal assessment [11–13]. Fetal fraction was initially estimated to be 3–6% during the early stages of the pregnancy [11] but is now known that the median fetal DNA fraction is about 14.5% of the cfDNA (cell free DNA) in the maternal circulation at the end of the first trimester [14]. The low percentage of fetal DNA in the maternal plasma represents the major challenge for the development of non-invasive diagnostic tests.

Up until a few years ago, in order to differentiate and detect the fetal-derived sequences from the maternal background, the most informative targets were based on absolute discriminative genetic markers, such as Y-chromosome-specific loci or paternally-inherited polymorphic loci that are either absent or different from the maternal genome [15–18]. However, in practice these early types of fetal markers are associated with certain limitations. First of all, they can only be used in pregnancies with male fetuses and second, they require the prior knowledge of the polymorphic status of the parents [19]. Consequently, it was necessary to develop a type of marker which allows confident differentiation of the fetus and the mother, and yet is independent of the gender or polymorphic status of the fetuses. A promising approach towards this end is the investigation of epigenetic differences between fetal and maternal DNA [19].

Epigenetic phenomena involve molecular changes that affect gene expression, and hence, alter the phenotype, without changing the DNA sequence context. DNA methylation is one of the best characterized epigenetic processes [20, 21]. It plays an important role in biological processes, such as cell differentiation and development. In addition, DNA methylation has a key role in carcinogenesis, with tumor-specific DNA methylation patterns in the plasma of cancer patients. Taking the above into account, it is quite likely to be able to identify fetal-specific markers from cffDNA in maternal circulation [4]. In 2002, the first differentially methylated region (DMR) between the fetal and the maternal cfDNA was discovered [22]. Since then, several studies have demonstrated the presence of a number of DMRs that distinguish fetal from maternal cfDNA [23–26]. The most widely used methods to study DNA methylation are sodium bisulfite treatment, methylation-sensitive restriction enzymes and methylated DNA immunoprecipitation (MeDIP).

Bisulfite conversion, which was developed by Frommer et al., remains the gold standard in DNA methylation analysis because it provides a qualitative, quantitative and efficient approach to identify 5-methylcytosine at single base-pair resolution [27]. Treatment with sodium bisulfite converts unmethylated cytosines to uracils which become thymines in the subsequent PCR amplification whereas methylated cytosines remain unchanged [28]. This results in the conversion of the usually undetectable epigenetic information into detectable sequence information at base pair resolution. The actual methylation status can be determined either by direct sequencing after PCR amplification or by cloning sequencing. Compared with other DNA methylation approaches bisulfite-based DNA methylation analysis has more quantitative accuracy, detection sensitivity, high efficiency and a wide spectrum of sample analysis [29].

The group of Dennis Lo applied genome wide bisulfite sequencing to analyze the fetal methylome non-invasively and serially [30]. In addition, sodium bisulfite was used in combination with digital PCR and/or mass spectrometry analysis for the quantification of cffDNA in maternal plasma [31]. As it was shown, the quantification of cffDNA can be facilitated by the combination of bisulfite conversion with the identification of SNPs within DMRs [24, 26, 32].

However, bisulfite sequencing has an important limitation. Due to the aggressive chemical conditions (low pH and high temperature) required to deaminate the unmethylated cytosines, there is a great extend of DNA degradation that can reach the level of 90% [28]. On the other hand, less aggressive treatments carry the risk of not converting efficiently all unmethylated cytosines, which results in an overestimation of methylation levels [33]. Therefore, it is very important and quite challenging to fine tune all the parameters of bisulfite treatment in order to achieve the best possible outcome. Several commercially available kits are trying to address these very issues.

The aim of this study was to compare the performance of four different kits for bisulfite conversion with respect to conversion efficiency, DNA degradation and conversion specificity. The kits compared were the Premium Bisulfite kit (Cat. No. C02030030, Diagenode), the EpiTect Bisulfite kit (Cat. No. 59104, Qiagen), the MethylEdge Bisulfite Conversion System (Cat. No. N1301, Promega) and the BisulFlash DNA Modification kit (Cat. No. 1026, Epigentek). These kits were chosen because they are widely used according to the literature. The performance was tested by using fully methylated and fully unmethylated λ-DNA controls in a series of spikes, by means of Sanger sequencing, Next-Generation Sequencing (NGS), gel electrophoresis and fluorometry. After identifying the most appropriate kit, we went on testing the methylation level at base pair resolution of two of our previously published DMRs [23] by forming serial spikes of DNA from CVS into DNA from whole blood of a non-pregnant woman starting from 50% and going as low as 3%. This spiking set-up in principle mimics the circulating free DNA in the blood of a pregnant woman.

## Materials and Methods

### Ethics statement

The study has been approved by the Cyprus National Bioethics Committee. Informed consent (written) was obtained from the two donors.

### Generation of λ-DNA fragments (unmethylated control)

Two λ-DNA fragments were synthesized by PCR. PCR was performed in a total volume of 25 μl containing 1.5 U AmpliTaq Gold Polymerase; 1x PCR reaction buffer containing 15 mM MgCl_2_; 0.2 mM each dNTPs; 5 pmol of each forward and reverse primers and 100 ng lambda genomic DNA. Reactions were incubated in a thermal cycler under the following conditions: a) 94°C for 2 min, b) 32 cycles of 94°C for 20 sec, Tm°C for 30 sec and 72°C for 60 sec and c) 72°C for 5 min, where Tm°C is the annealing temperature. PCR products were purified using the QIAquick PCR Purification kit according to manufacturer’s instructions and eluted in 30 μl of molecular biology grade water. The obtained amplicons were visualized on a 2% agarose gel under UV light and correct sizes were confirmed.

### In vitro methylation of λ-DNA fragments (methylated control)

Each of the two λ-DNA fragments was divided into two aliquots. Since PCR amplification removes all the methyl groups present in the template DNA, one of these two aliquots was used as the fully unmethylated λ-DNA control. The other aliquot was in vitro methylated by incubating 100 ng λ-DNA with 2 μl 10x NEBuffer2, 4 U SssI methyltransferase and 160 μM S-adenosylmethionine (SAM) in a total volume of 20 μl at 37°C for 3 h, then 65°C for 20 min. DNA was purified using the QIAquick PCR Purification kit and eluted in 30 μl of molecular biology grade water. To assess the level of methylation, each amplicon was designed to contain at least one 5’-ACGT-3’ motif which is a recognition site of the CpG methylation-sensitive restriction endonuclease, Hpy-CH4IV. Therefore, cleavage of the motif only occurs in the absence of CpG methylation. The digestion was performed by incubating 100 ng of the purified methylated PCR products with 2.5 μl 10x NEBuffer 1 and 10 U HpyCH4IV in a total volume of 25 μl for 1 h at 37°C and 20 min at 65°C. DNA was purified using the QIAquick PCR Purification kit and eluted in 30 μl of molecular biology grade water. The products of digestion were visualized on a 2% agarose gel under UV light and correct sizes were confirmed.

### Spikes preparation and bisulfite treatment

Concentrations of the methylated and unmethylated λ-DNA fragments were quantified with the Qubit fluorometer (Invitrogen) as per manufacturer's instructions. Spikes were prepared for downstream analysis with Sanger sequencing and NGS (starting amount of DNA: 200 ng). For each of the two λ-DNA fragments, 100 ng was used for the spikes of the Sanger sequencing and 25 ng was used for the spikes of the NGS. In addition, for the spikes of the NGS experiment human genomic DNA was used from WB and CVS in a total amount of 150 ng. Spikes were prepared for Sanger sequencing as shown in Table 1 and for NGS as shown in Table 2. All samples were prepared in triplicates.

**Table 1:** PCR amplicons that were used for bisulfite conversion and sanger sequencing.

	Premium Bisulfite kit		EpiTect Bisulfite kit		MethylEdge Bisulfite Conversion System		BisulFlash DNA Modification kit	
	Unmethylated λ-DNA	Methylated λ-DNA	Unmethylated λ-DNA	Methylated λ-DNA	Unmethylated λ-DNA	Methylated λ-DNA	Unmethylated λ-DNA	Methylated λ-DNA
spike 1	100%	0%	100%	0%	100%	0%	100%	0%
spike 2	75%	25%	75%	25%	75%	25%	75%	25%
spike 3	50%	50%	50%	50%	50%	50%	50%	50%
spike 4	0%	100%	0%	100%	0%	100%	0%	100%

Spikes were prepared for sanger sequencing as shown using methylated and unmethylated λ-DNA fragments.

**Table 2:** PCR amplicons that were used for bisulfite conversion and NGS.

	Premium Bisulfite kit				EpiTect Bisulfite kit				MethylEdge Bisulfite Conversion System				BisulFlash DNA Modification kit			
	Unmethylated λ-DNA (%)	Methylated λ-DNA (%)	WB DNA (%)	CVS DNA (%)	Unmethylated λ-DNA (%)	Methylated λ-DNA (%)	WB DNA (%)	CVS DNA (%)	Unmethylated λ-DNA (%)	Methylated λ-DNA (%)	WB DNA (%)	CVS DNA (%)	Unmethylated λ-DNA (%)	Methylated λ-DNA (%)	WB DNA (%)	CVS DNA (%)
sp. 1	100	0	100	0	100	0	100	0	100	0	100	0	100	0	100	0
sp. 2	97	3	97	3	97	3	9	3	97	3	97	3	97	3	97	3
sp. 3	95	5	95	5	95	5	95	5	95	5	95	5	95	5	95	5
sp. 4	93	7	93	7	93	7	93	7	93	7	93	7	93	7	93	7
sp. 5	90	10	90	10	90	10	90	10	90	10	90	10	90	10	90	10
sp. 6	75	25	75	25	75	25	75	25	75	25	75	25	75	25	75	25
sp. 7	50	50	50	50	50	50	50	50	50	50	50	50	50	5	50	50
sp. 8	0	100	0	100	0	100	0	100	0	100	0	100	0	100	0	100

Spikes were prepared for NGS as shown using methylated and unmethylated λ-DNA fragments and human genomic DNA from WB and CVS.

Bisulfite conversion and subsequent purification was performed according to the respective protocols. The kits that were used were: the Premium Bisulfite kit (Cat. No. C02030030, Diagenode), the EpiTect Bisulfite kit (Cat. No. 59104, Qiagen), the MethylEdge Bisulfite Conversion System (Cat. No. N1301, Promega) and the BisulFlash DNA Modification kit (Cat. No. 1026, Epigentek). For the determination of the DNA yield after bisulfite conversion, the Qubit fluorometer (Invitrogen) was used according to manufacturer's instructions.

### Bisulfite specific PCR

Bisulfite specific primers that are complementary to the sense strand of the converted DNA were designed using the Methyl Primer Express Software v1.0. The primers were designed so that they didn’t contain any CpGs in order to facilitate their binding to both methylated and unmethylated sequences.

PCR was performed in a total volume of 25 μl containing 1.5 U AmpliTaq Gold Polymerase; 1x PCR reaction buffer containing 15 mM MgCl_2_; 0.2 mM each dNTPs; 5 pmol each forward and reverse primers and 1 μl of converted DNA. Reactions were incubated in a thermal cycler under the following conditions: a) 94°C for 5 min, b) 40 cycles of 94°C for 30 sec, Tm°C for 40 sec and 72°C for 45 sec and c) 72°C for 7 min, where Tm°C is the annealing temperature. The amplicons were visualized on a 2% agarose gel under UV light and correct sizes were confirmed.

### Sanger sequencing

ExoSAP-IT was used for the clean-up of the λ-DNA PCR products (410bp and 309bp) according to the manufacturer’s instructions (Affymetrix, Santa Clara, CA, USA). The two λ-DNA PCR products were covered both by forward and reverse strand sequencing using the BigDye Terminator v1.1 Cycle Sequencing Kit (Applied Biosystems, Carlsbad, CA, USA) and the ABI 3130*xl* Genetic Analyzer (Applied Biosystems) according to the manufacturer's protocol. The sequencing primers were the same as those used for the PCR amplification. Sequence traces were automatically compared with the *in silico* converted λ-DNA sequences using the ABI SeqScape software (Applied Biosystems). Chromatograph traces (.abi) were analyzed using the ESME analysis software [34].

### Next-Generation Sequencing and Alignment

The individual PCR products of the two λ-DNA controls and the two DMRs were purified using the QIAquick PCR Purification kit and were quantified using the Qubit fluorometer (Invitrogen) according to manufacturer's instructions. The two amplicons of the λ-DNA controls and the two DMRs coming from the same conversion were pooled together equimolarly (in some cases the concentration of the PCR product was very low and therefore these products were excluded from the library preparation). The pooled amplicons were blunt ended, ligated to Illumina sequencing adapters and amplified by 10 cycles with indexing primers. The 96 libraries were analyzed on 2200 TapeStation and quantified by the Qubit fluorometer. They were then pooled equimolarly and sequenced on the Illumina MiSeq sequencer (as 25% spike in genomic DNA with 1% PhiX), with paired-end sequencing reads of 2 x 251bp. Data was demultiplexed on the MiSeq instrument automatically, and a zipped FASTQ file was generated per sample. The raw FASTQ datasets were accessed through BaseSpace beta (basespace.illumina.com). The raw reads in the FASTQ files were aligned to their corresponding reference genomes using BSMAP, a general-purpose bisulfite mapping software [35] which maps the reads to bisulfite converted and non-bisulfite converted reference genomes.

## Results

### Conversion efficiency

**Fig 1:**
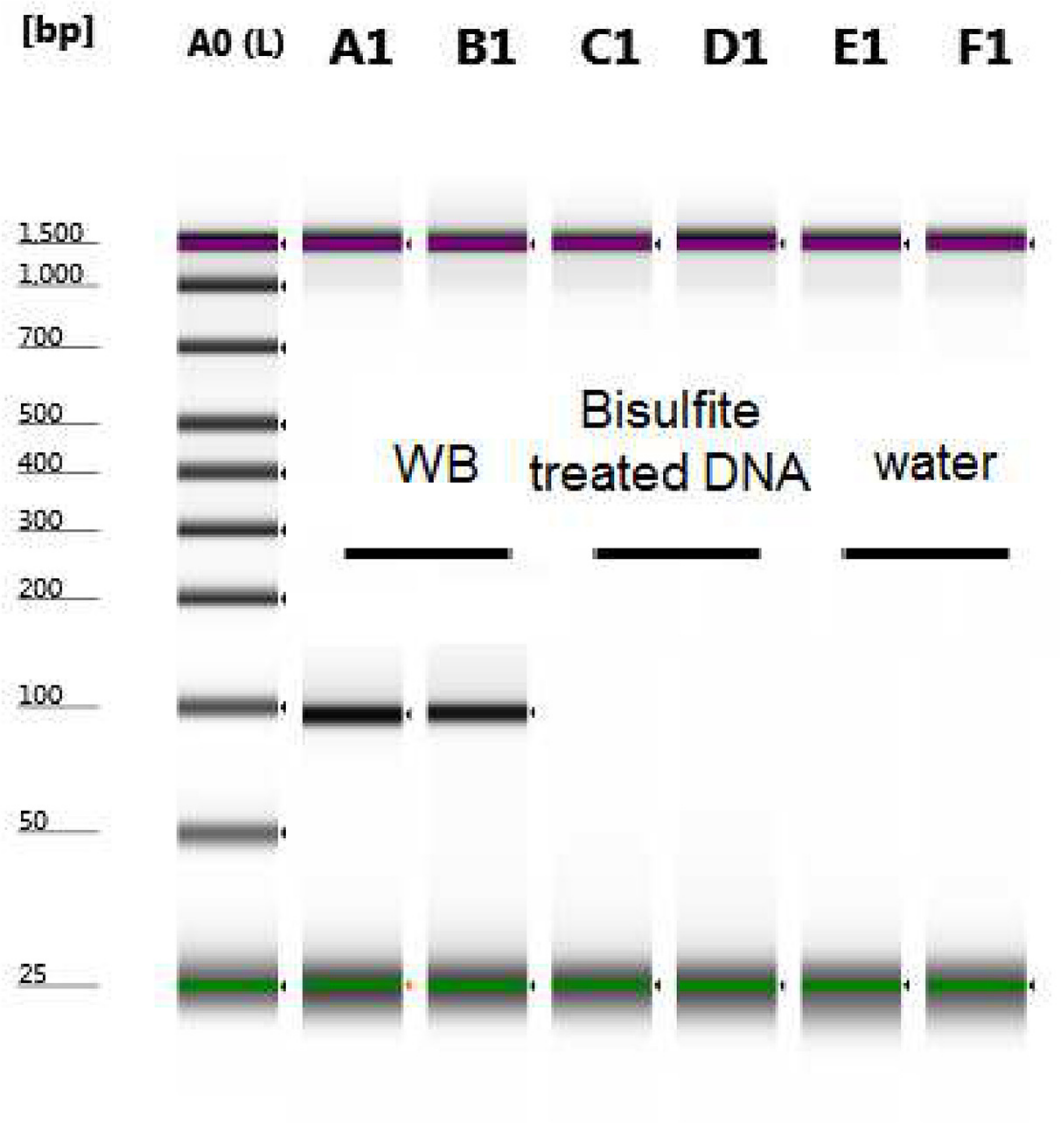
PCR amplification using regular primers. As a quality control step, the bisulfite converted DNA was amplified with regular non-bisulfite specific primers that bind to the unconverted DNA sequence. Unconverted whole blood DNA was used as a positive control and water as negative control. Shown is a picture of the gel run in Agilent TapeStation 2200, where there is no PCR product in the 2 replicates with the bisulfite treated DNA suggesting that all DNA was converted successfully.

As a quality control (QC) step, we used the bisulfite converted DNA to perform PCR with regular non-bisulfite specific primers in order to amplify any unconverted product and thus make an initial assessment of the conversion efficiency. By using primers that are complementary to the non-converted sequence of one of our DMRs (F:TGAATCAGTTCACCGACAGC, R:GAAACAACCTGGCCATTCTC), it was shown that no PCR product was detected, suggesting full DNA conversion (Fig 1).

**Fig 2:**
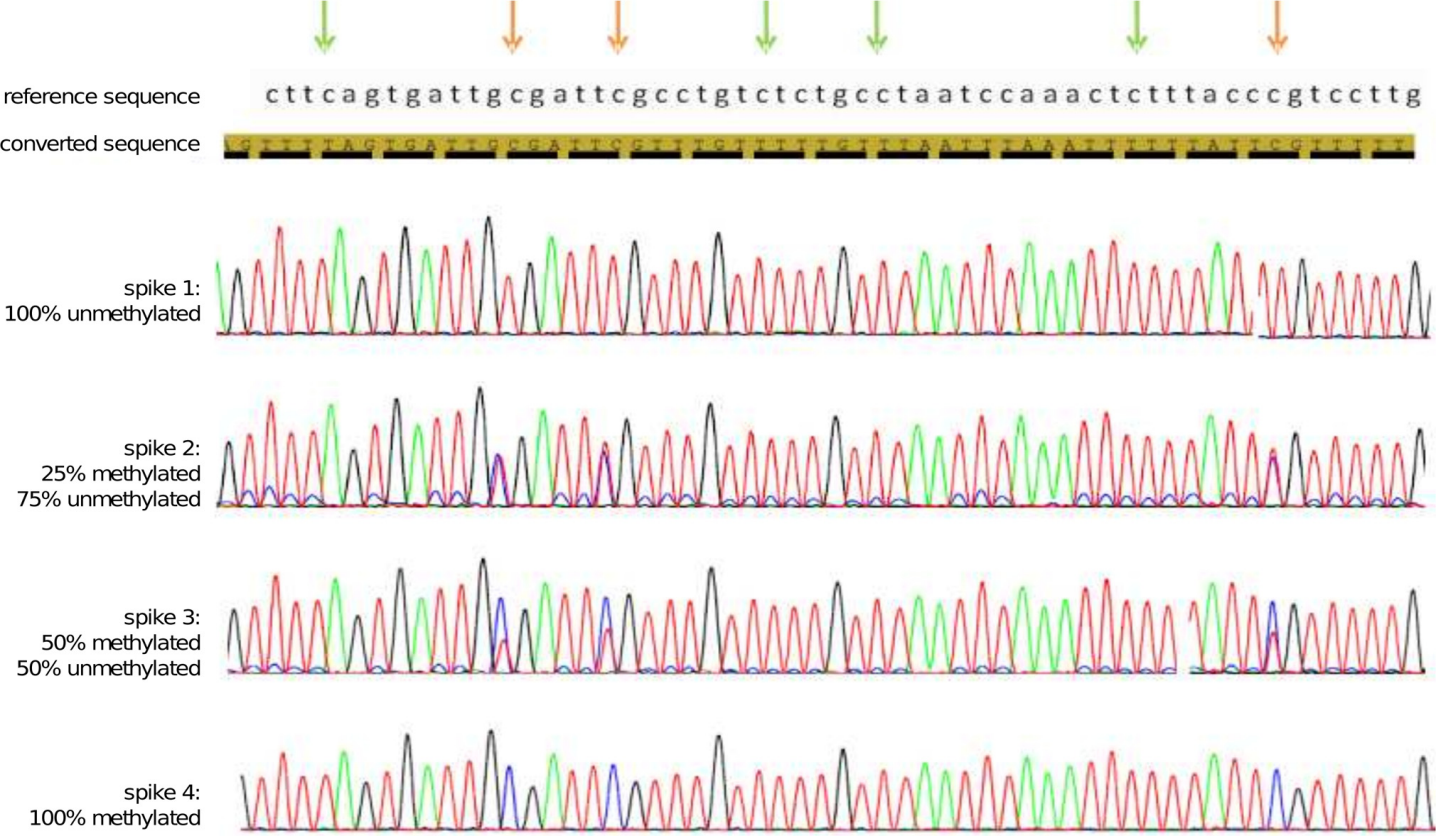
Bisulfite sanger sequencing of the λ-DNA methylation controls. A section of the sequencing trace for all spikes is presented. The reference sequence and the in-silico converted sequences are shown on top. Green arrows indicate non-CpG cytosines (blue) that are all converted to thymines (red) in all spikes. Orange arrows indicate CpG cytosines that are fully converted in spike 1, partially converted in spikes 2 and 3 and not converted in spike 4.

In order to accurately estimate conversion efficiency, PCR amplicons were sequenced with the Sanger method and on the Illumina MiSeq benchtop sequencer. The conversion efficiency can only be assessed using the non-CpG cytosines since they are unmethylated and should all be converted to thymines. By aligning our Sanger sequencing data to the *in silico* converted λ-DNA sequences using the ABI SeqScape software (Applied Biosystems) we were able to detect 100% conversion efficiency for all our λ-DNA spikes treated with all the kits (Fig 2).

However, the sensitivity of Sanger sequencing for showing small percentages of alleles is limited, therefore we further analyzed the conversion efficiency of each kit with deep sequencing on an Illumina MiSeq platform. The MiSeq run information is summarized in S1 Table. The number of reads mapped on the target sequences and passed the quality filter for the 96 libraries of the two λ-DNA controls and the two DMRs is shown in S2 Table. For the calculation of the conversion efficiency the λ-DNA control 1 was used since this amplicon was present in all the libraries.

**Fig 3:**
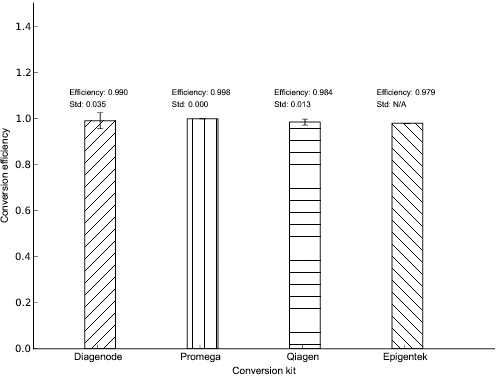
Bisulfite conversion efficiency of the different kits. The conversion efficiency of the unmethylated cytosines to thymines was calculated per kit. All the non-CpG cytosines of all the 24 libraries of the Lambda control 1 were used for the calculation. Conversion efficiency of the Premium Bisulfite kit (Diagenode) was 99.0±0.035%, of the MethylEdge Bisulfite Conversion System (Promega) was 99.8±0.000%, of the EpiTect Bisulfite kit (Qiagen) was 98.4±0.013% and of the BisulFlash DNA Modification kit (Epigentek) was 97.9%. Error bars = SD.

By using the BSMAP software we aligned the paired reads to the lambda reference genome. To calculate the conversion efficiency of each kit, we utilised the counts of the non-CpG cytosines (C) and thymines (T) found on the Watson strand. These non-CpG Cs were expected to be converted to thymines upon completion of the bisulfite protocol because of the lack of methylation in these sites. Thus, we calculated the percentage by dividing the number of non-CpG C's by the sum of non-CpG C's and T's and then multiplying by 100, which should give 0% after complete conversion. The average of the values of all the sites from all the 24 libraries of each kit was calculated and the methylation percentage for each kit was determined. By subtracting this from 100%, the conversion efficiency was obtained. As shown in Fig 3, the conversion efficiency of the Premium Bisulfite kit (Diagenode) was 99%, of the MethylEdge Bisulfite Conversion System (Promega) was 99.8%, of the EpiTect Bisulfite kit (Qiagen) was 98.4% and of the BisulFlash DNA Modification kit (Epigentek) was 97.9%.

### DNA yield and degradation

**Fig 4:**
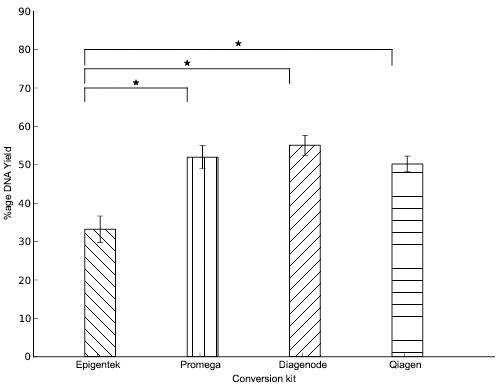
DNA degradation. DNA recovery after bisulfite treatment was determined with the Qubit fluorometer. DNA recovery with the Epigentek kit was 33.2% ± 3.4%, with the Promega kit was 52% ± 3%, with the Diagenode kit was 55% ± 2.6% and with the Qiagen kit was 50.2% ± 2%. The BisulFlash DNA Modification kit was shown to have a significantly decreased average yield with respect to all other kits (FWER < 0.05, f-value = 10.5, p*-val = 2*.*8E-5)*. Shown are the mean values of n = 12. Error bars = SD.

In order to compare the DNA yield and hence, the degradation effect of each of the four different kits we used the Qubit fluorometer (Invitrogen) to determine the bisulfite-treated DNA concentration. All the bisulfite-treated DNA samples that were going to be tested with sanger sequencing were quantified with the Qubit fluorometer immediately after the conversion. The percentage of DNA yield was calculated for each sample using the ratio of the total DNA amount after the treatment to the total DNA amount before the treatment (200ng) and multiplied by 100. For each kit the average yield was calculated from the twelve samples (4 spikes x 3 replicates). As shown in Fig 4, the highest yield was observed when using the Premium Bisulfite kit (55% ± 2.6%) followed by the MethylEdge Bisulfite Conversion System (52% ± 3%) while the lowest yield when using the BisulFlash DNA Modification kit (33.2% ± 3.4%). An ANOVA test accompanied by post-hoc analysis provided evidence that the latter kit has a significantly different average yield with respect to all other kits (FWER < 0.05, f-value = 10.5, p*-val = 2*.*8E-5)*.

### Conversion specificity

The specificity of the conversion of unmethylated cytosines to uracils was studied by using known spikes of artificially methylated λ-DNA into a fully unmethylated λ-DNA. The percentage of methylated DNA in unmethylated DNA was 0%, 25%, 50% and 100% for the converted amplicons that were sequenced with Sanger sequencing.

**Fig 5:**
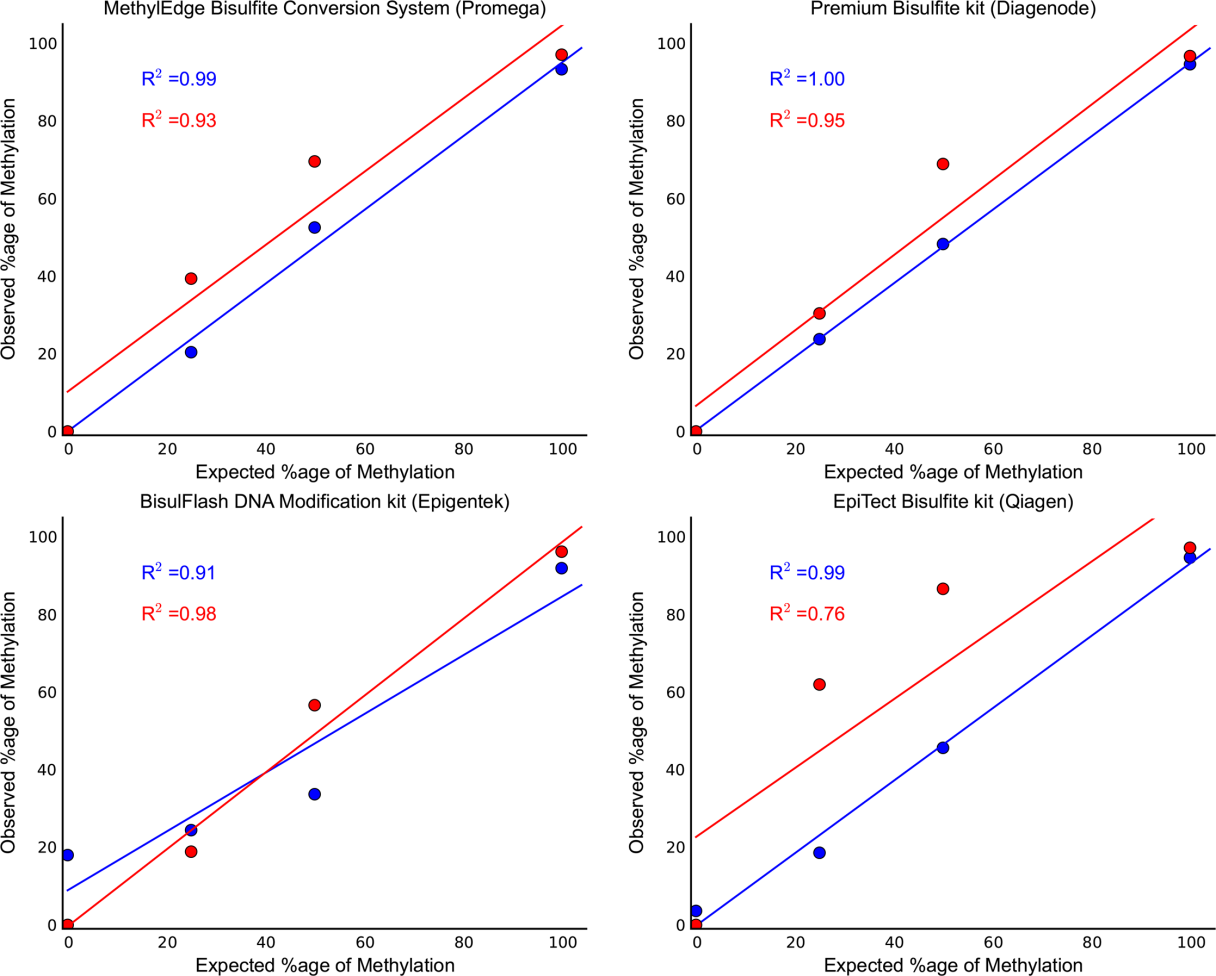
Bisulfite amplicon sanger sequencing. Standard curves were generated by sanger methylation quantification of known methylation level controls. The expected percentage of methylation was plotted versus the observed one. Points represent the mean methylation level of the fifteen and eleven CpG sites for the two regions, respectively, as analyzed with Sanger/ESME (blue line = Lambda control 1, red line = Lambda control 2). Both λ-DNA controls were able to fit linear lines with R^2^ values as presented. The correlation coefficients are higher in the data generated from the Premium Bisulfite kit (Diagenode) and the MethylEdge Bisulfite Conversion System (Promega). [P value for i) Lambda control 2 (Qiagen) = 0.1309, ii) rest of samples < 0.05].

Standard curves were generated for each conversion kit by taking the mean methylation level of the CpG sites across the amplified region of the two λ-DNA controls as analyzed with Sanger/ESME. These were fifteen and eleven CpG sites for the two regions, respectively. Fig 5 shows the observed methylation levels in correlation with the expected methylation levels. Both λ-DNA controls were able to fit linear lines with R^2^ values as shown in Fig 5. The higher correlation coefficients were generated from the Premium Bisulfite kit (Diagenode) and the MethylEdge Bisulfite Conversion System (Promega) suggesting more accurate methylation quantitation [P value for i) Lambda control 2 (Qiagen) = 0.1309, ii) rest of samples < 0.05].

By analyzing the electropherogram of the Sanger sequencing data it was demonstrated that all the non-CpG cytosines were fully converted to thymines and the CpG cytosines were fully, partially or not converted at all to thymines, depending if the spike of λ-DNA is 0%, 25%, 50% or 100% methylated (Fig 2). Moreover, it was observed that there is a potential preferential amplification of the methylated over the unmethylated DNA. This was indicated in the 50% and the 25% methylated spikes, by the fact that the fluorescent signal of the cytosine compared to the thymine at the CpG sites was stronger than what was expected.

Based on the results obtained from the comparison of the four kits regarding the conversion efficiency and the degradation effect together with the conversion specificity from the Sanger sequencing, we selected the Premium Bisulfite kit (Diagenode) and the MethylEdge Bisulfite Conversion System (Promega) as the two kits with the best performance.

**Fig 6:**
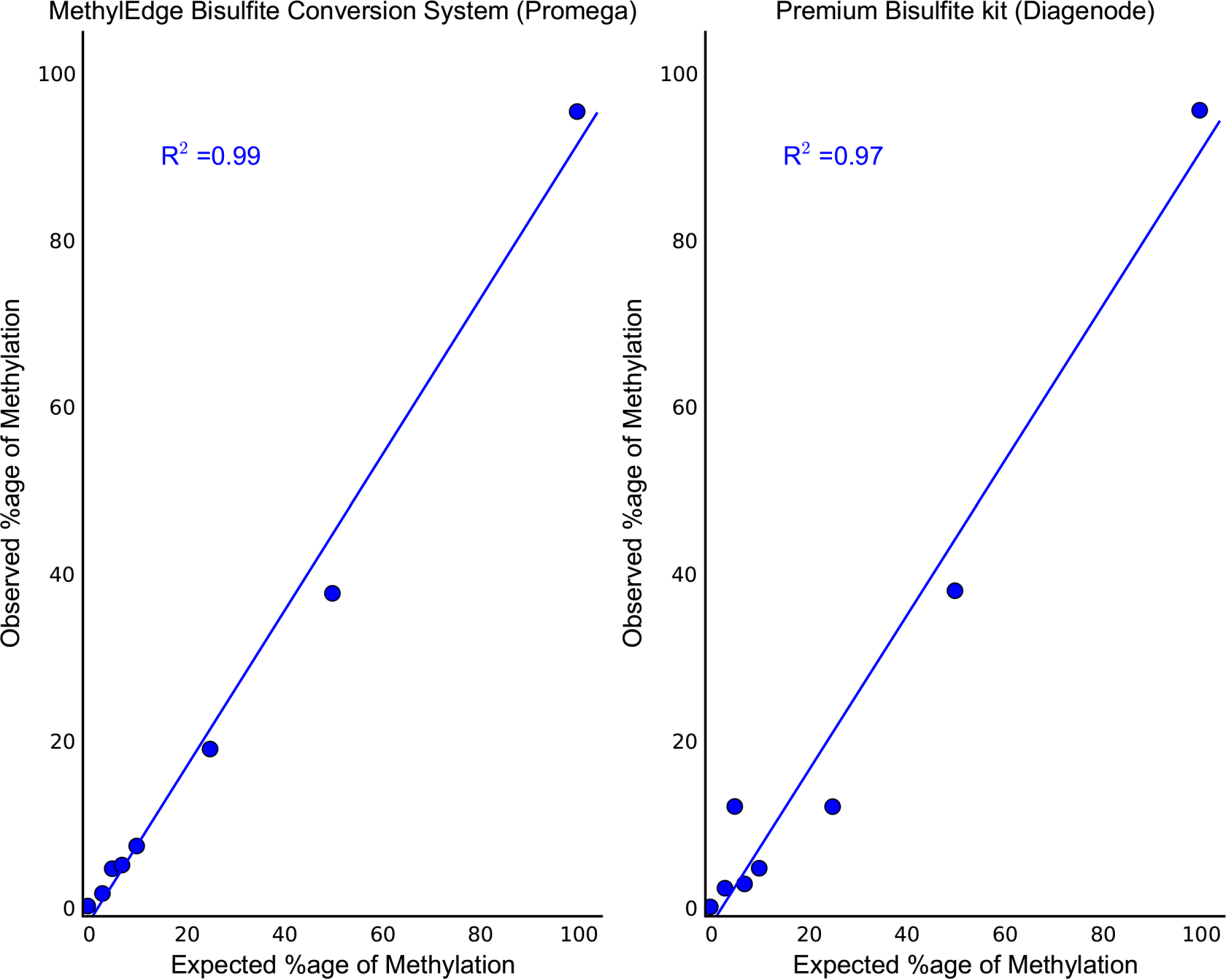
Bisulfite amplicon next generation sequencing. Standard curves were generated by next generation sequencing methylation quantification of known methylation level controls. The expected percentage of methylation was plotted versus the observed one. Points represent the mean methylation level of the fifteen CpG sites of the region as analyzed with BSMAP. Lambda-DNA control 1 was able to fit linear lines with both kits with R^2^ values as presented [P value for both samples < 0.05].

These two kits were further analyzed regarding their conversion specificity with bisulfite sequencing on an Illumina MiSeq platform. The spikes of the two methylated λ-DNA controls into the unmethylated ones were 0%, 3%, 5%, 7%, 10%, 25%, 50% and 100%. These were used to generate standard curves for the two conversion kits by taking the mean methylation level of the CpG sites across the λ-DNA control as analyzed with BSMAP. Fig 6 shows the observed methylation levels in correlation with the expected methylation levels. The λ-DNA control 1 was able to fit linear lines with R^2^ = 0.99 and 0.97 for the Promega and the Diagenode kit, respectively, indicating that both kits performed very well [P value for both samples < 0.05]. Though the difference in the performance of the two kits was marginal, only one kit was selected for downstream experiments involving DMR analysis. Since the two kits performed similarly, the only selection criterion to distinguish between the two kits was the small difference in the correlation coefficient. Therefore, the MethylEdge Bisulfite Conversion System (Promega), was selected to be used for further analysis of our DMRs.

**Fig 7:**
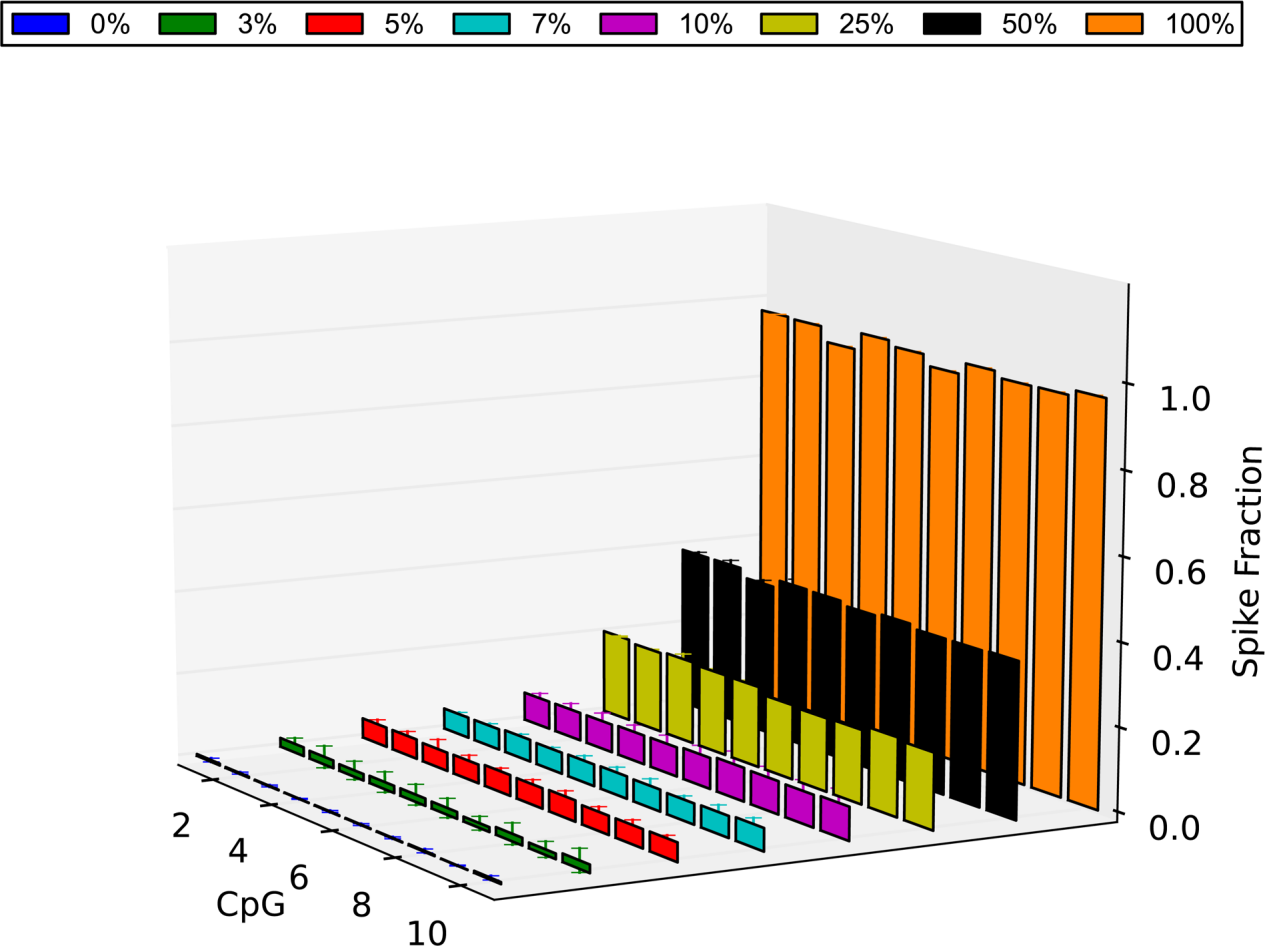
Methylation level of λ-DNA control as determined by NGS. The methylation level of all the CpG sites of the amplified region of the λ-DNA control 2 is plotted for all the spikes. Shown is the average value of each CpG in each spike as was calculated from 3 technical replicates (methylated λ-DNA 0% = blue, 3% = green, 5% = red, 7% = light blue, 10% = purple, 25% = yellow, 50% = black, 100% = orange). In order to calculate statistical significance of the methylation level of each spike, we created a dataset of average performance for each spike group and we compared these average performance datasets with the 100% unmethylated average performance set using a t-test. It was demonstrated that all the small-percentage spikes showed significantly higher methylation levels than the 0% methylated group [3% (P = 2.704e-11), 5% (P = 1.685e-21), 7% (P = 1.181e-23), 10% (P = 2.991e-19)]. Errors bars = SD

Consequently, by calculating the average methylation value of each CpG in each spike obtained from the 3 technical replicates, the methylation level of all CpG sites was investigated (Fig 7). As it is shown, the observed methylation level of each CpG is very close to the expected one in all spikes with a slight underestimation of methylation levels. As reported by others, this is likely due to the methylated DNA containing secondary structures in the template causing a reduction in PCR efficiency compared to unmethylated sequences [36]. In order to estimate statistical significance, we used the mean methylation level of all the CpGs in each spike to create a dataset of average methylation levels for each spike group. We then compared these mean methylation level datasets with the mean methylation level of the group of the 0% methylated λ-DNA using a t-test. It was demonstrated that all spike groups showed significant differences between their methylation level and the fully unmethylated group. More importantly, all the small-percentage spikes showed significantly higher methylation levels than the 0% methylated group [3% (P = 2.704e-11), 5% (P = 1.685e-21), 7% (P = 1.181e-23), 10% (P = 2.991e-19)]. This suggests that using this bisulfite amplicon sequencing approach, discrimination can occur between methylated and unmethylated DNA even when the methylated DNA fraction is as low as 3%.

### DMRs methylation analysis using the selected kit

After evaluating the different kits and proving that the MethylEdge Bisulfite Conversion System (Promega) had the highest performance of all, we took one step forward and we used this kit to analyze the methylation level of our DMRs. Two of our previously identified DMRs (EP6 and EP10) [23] were investigated with bisulfite/NGS and their methylation level was studied for the first time at base pair resolution. The same percentages that were used as spikes for the λ-DNA controls were also used for the DMRs using one CVS sample spiked into whole blood DNA of a non-pregnant woman (Table 2).

**Fig 8:**
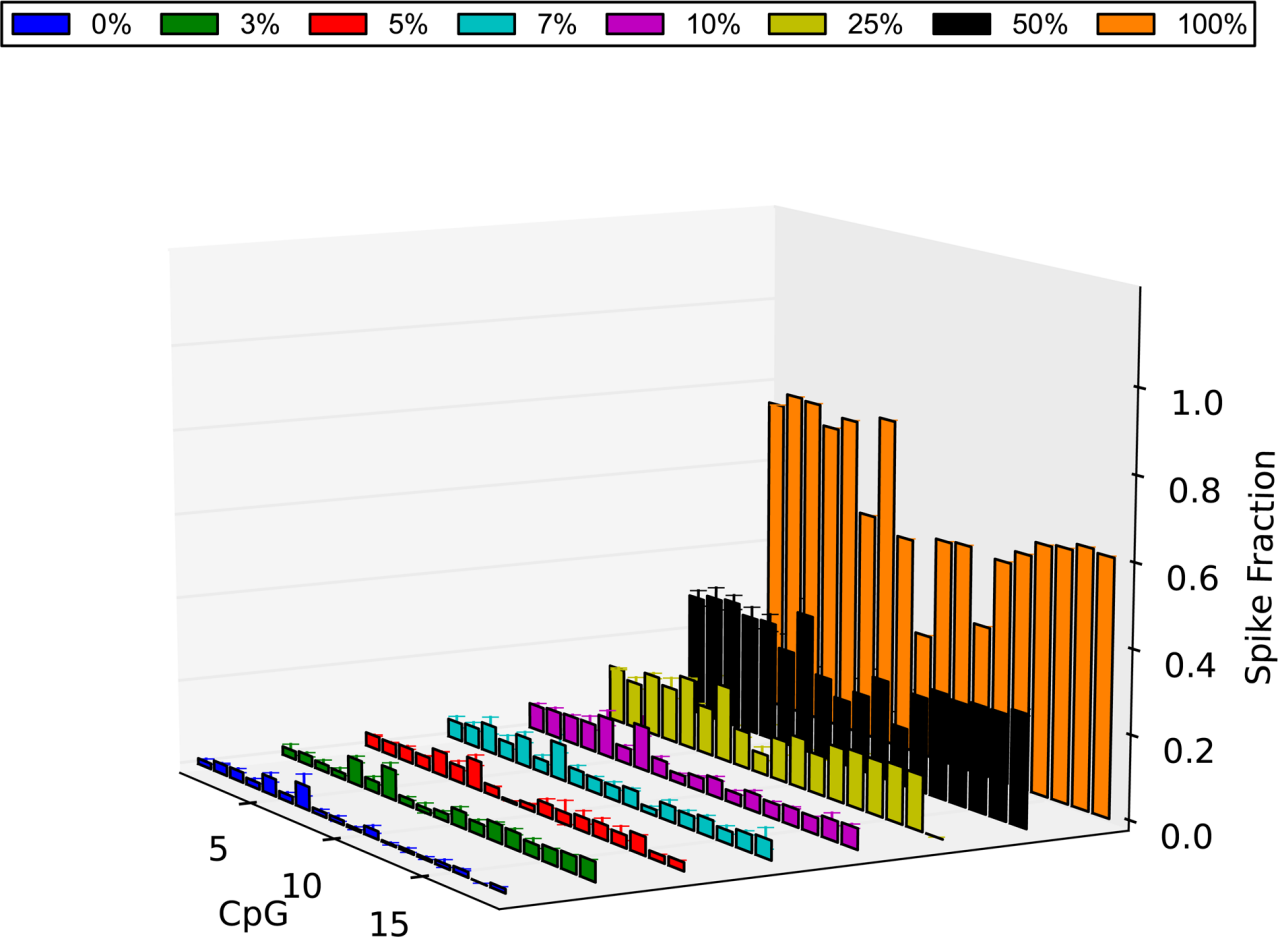
Methylation level of the EP6 DMR as determined by NGS. The methylation level of all the CpG sites of the amplified region of the EP6 DMR is plotted for all the spikes. Shown is the average value of each CpG in each spike as was calculated from 3 technical replicates (CVS 0% = blue, 3% = green, 5% = red, 7% = light blue, 10% = purple, 25% = yellow, 50% = black, 100% = orange). In order to calculate statistical significance of the methylation level of each spike, we created a dataset of average performance for each spike group and we compared these average performance datasets with the 0% CVS average performance set using a t-test. It was demonstrated that all the small spikes showed significantly higher methylation levels than the 0% CVS group [3% (P = 0.004), 5% (P = 0.004), 7% (P = 1.107e-05), 10% (P = 4.458e-06)]. Error bars = SD.

**Fig 9:**
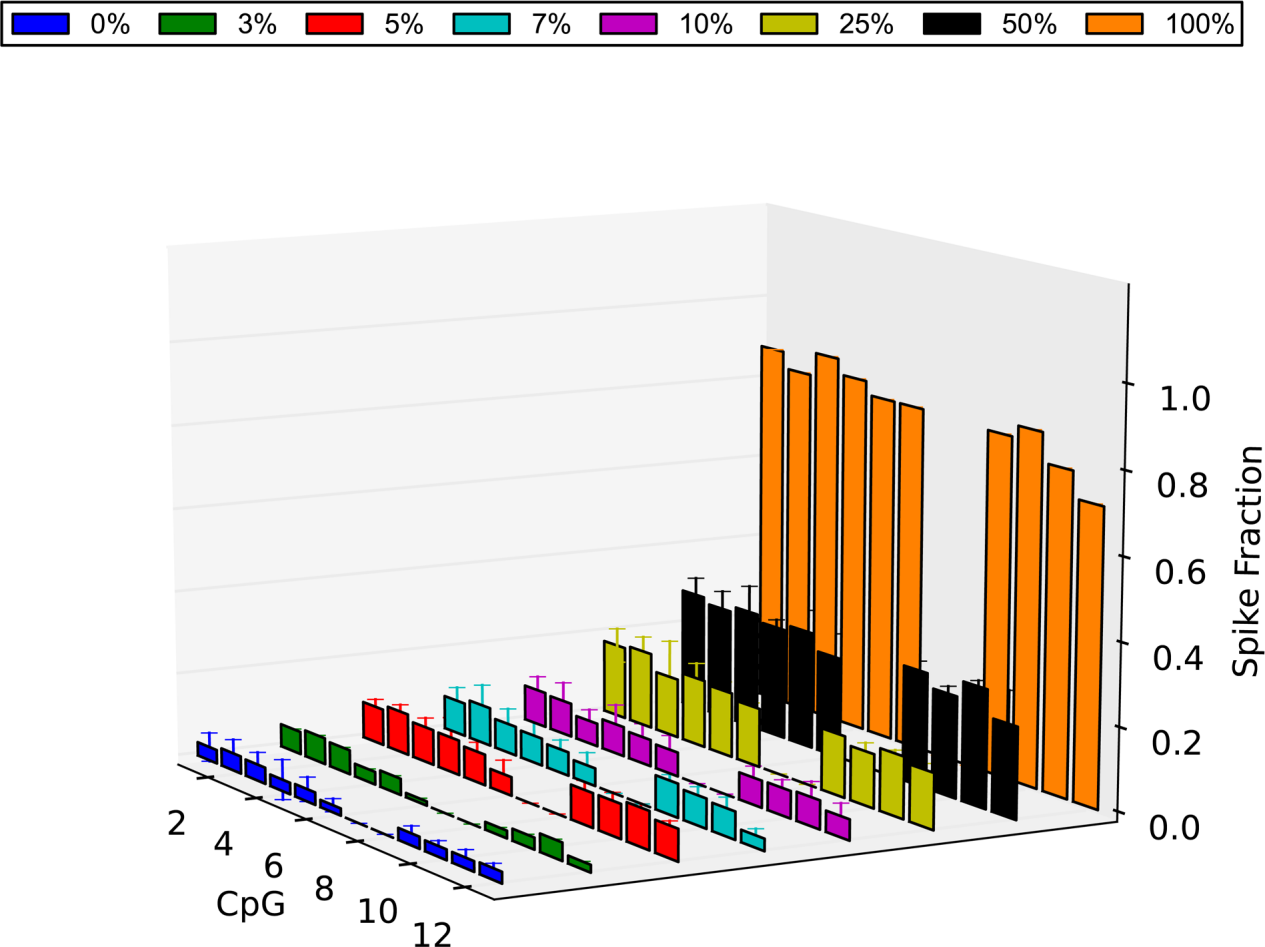
Methylation level of the EP10 DMR as determined by NGS. The methylation level of all the CpG sites of the amplified region of the EP10 DMR is plotted for all the spikes. Shown is the average value of each CpG in each spike as was calculated from 3 technical replicates (CVS 0% = blue, 3% = green, 5% = red, 7% = light blue, 10% = purple, 25% = yellow, 50% = black, 100% = orange). In order to calculate statistical significance of the methylation level of each spike, we created a dataset of average performance for each spike group and we compared these average performance datasets with the 0% CVS average performance set using a t-test. It was demonstrated that the small spikes showed significantly higher methylation levels than the 0% CVS group [5% (P = 7.239e-09), 7% (P = 6.612e-06), 10% (P = 1.068e-07)]. Errors bars = SD

The average value of each CpG in each spike obtained from the 3 technical replicates was calculated and the methylation level of all CpG sites was determined as previously explained. Our DMRs were confirmed and investigated at single base resolution. From the experiments, EP6 showed high methylation level in some CpG sites (~80%), moderate in others (50–60%) and low in 2 CpGs (35–40%) in the CVS and low methylation level in WB (<10%) (Fig 8). EP10 shows more consistency with high methylation in all CpG sites in the CVS (>75%) and low in the WB (<10%) (Fig 9). In order to estimate statistical significance, we used the same principle as before. It was demonstrated that for EP6 all the small-percentage spikes of CVS showed significantly higher methylation levels than the 0% CVS group [3% (P = 0.004), 5% (P = 0.004), 7% (P = 1.107e-05), 10% (P = 4.458e-06)]. For EP10 there was a significant difference between the methylation levels of the 0% CVS group and the 5% (P = 7.239e-09), the 7% (P = 6.612e-06) and the 10% (P = 1.068e-07) group.

## Discussion

DNA methylation analysis has undergone a major technological revolution in recent years. Especially, bisulfite conversion when coupled to NGS, has enabled genome wide methylation analysis in a high throughput manner at single base resolution with a quick turn-around time. Aggressive bisulfite treatment protocols (long incubation, high temperatures, high molarity of bisulfite) secure full conversion of cytosines to uracils, but DNA can be degraded to a degree that makes PCR amplification impossible. On the other hand, less aggressive treatments bear the risk of incomplete conversion and therefore can lead to overestimation of the methylation levels. Therefore, the challenge of the method is that the desired outcomes (conversion of unmethylated cytosines) positively correlate with the undesired side effects (DNA degradation and inappropriate conversion) and one should aim to find the best possible balance between the two. Several kits are commercially available for bisulfite conversion of DNA, each with their own advantages and disadvantages. One of the most important parameters of bisulfite conversion is the yield of DNA post treatment. It is crucial to be sufficiently high for downstream molecular analyses. Furthermore, the incomplete and inappropriate conversion during bisulfite treatment is another imperative factor and should be considered carefully. In this study we aimed to compare four different bisulfite conversion kits with regards to their conversion efficiency, DNA degradation and conversion specificity. The kits that were compared were the Premium Bisulfite kit (Cat. No. C02030030, Diagenode), the EpiTect Bisulfite kit (Cat. No. 59104, Qiagen), the MethylEdge Bisulfite Conversion System (Cat. No. N1301, Promega) and the BisulFlash DNA Modification kit (Cat. No. 1026, Epigentek).

A range of targeted bisulfite sequencing techniques emerged over the last decade and bisulfite sequencing is now actively employed in the field of cancer research, investigation of drugs metabolism as well as identification of epigenetic markers of different cell types [37–40]. The advantage of targeted compared to whole genome sequencing is the increase in the achieved sequence depth of the chosen areas of the genome in tandem with the reduction of the amount of generated data which leads to decreased processing time and costs. The principle of bisulfite amplicon sequencing was utilized in a study by Masser *et al*. in 2013, where they showed that methylated cytosine quantification can be accurate, rapid and cost-effective with base specificity [41].

Among targeted approaches for methylation analysis, bisulfite amplicon sequencing is suitable for the quantitative methylation analysis of specific genes or regions, particularly when a large number of samples is under investigation. This is because in bisulfite amplicon sequencing, multiple target regions in one sample and multiple samples can be sequenced jointly on one flow cell. In our case, 96 samples each consisting of 4 amplicons were multiplexed and sequenced on one single-lane flow cell. Compared to whole methylome studies that are useful for wide discovery purposes, bisulfite amplicon sequencing constitutes a means for answering hypothesis driven research questions or investigating specific regions previously identified in initial methylome studies [41].

Bisulfite treatment results in a fragmented single stranded mixture of nucleic acids that has features of DNA and RNA. The main cause of fragmentation has been identified to be the step of depurination [42]. High levels of DNA degradation decrease the number of DNA molecules, that are actually available for PCR amplification. The number of available molecules is also influenced by the length of the target amplicon [33]. Longer amplicons are less likely to amplify, merely because the possibility to find a single intact starting template decreases. When the DNA is fragmented and only a few available molecules are left, in addition to the failed PCR amplification, the results of PCR are no longer quantitative and present large variability when measured repeatedly [33]. All these negative side effects of bisulfite conversion on the integrity of DNA are crucial and therefore estimating DNA recovery after the conversion is a very important parameter in bisulfite sequencing. Here, we compared the DNA recovery of the four kits by measuring DNA concentration using the Qubit fluorometry. As can be seen in Fig 4, the DNA recovery ranged between 33.2% and 55%. All kits yielded DNA that was suitable for downstream PCR analysis (Fig 4).

It was suggested that the DNA sequence can affect conversion efficiency, since stable secondary structure elements formed in the DNA after denaturation can interfere with conversion [43, 44]. In the study of mammalian DNA methylation, where methylation essentially occurs only in CpG sites, all the non-CpG cytosines can be studied as an indicator for conversion efficiency [45] and this approach was followed here. The conversion efficiency of each kit was assessed by means of Sanger sequencing and 100% conversion efficiency of our λ-DNA controls was observed with all the kits (Fig 2).However, direct Sanger sequencing has the capacity to detect two bases at a specific site when the minor component is ≥15% [46]. Below 15% the cytosine signal may not be detected in a not completely converted cytosine site. Therefore, we sequenced the bisulfite converted DNA (Table 2) on an Illumina MiSeq platform, in order to overcome the reduced sensitivity of Sanger sequencing. The NGS data was analyzed using BSMAP which is a flexible and efficient general-purpose bisulfite sequence mapping program. It uses the positions of all cytosines in the reference sequences and implements an asymmetric C/T mapping: T in bisulfite converted reads can be mapped to either C or T in the reference but not vice versa. Additionally, BSMAP supports the detection of multiple CpG heterogeneous methylation patterns and C methylations that are not at a CpG site [35]. By taking into account the methylation level of all the non-CpG cytosines across the entire length of the lambda DNA control 1 in all the samples, we determined the conversion efficiency of each kit (Fig 3). The efficiencies ranged from 97.9% to 99.8% which is considered to be a sufficiently high conversion for all biological and clinical applications. As shown in Fig 3, the conversion efficiency of the Premium Bisulfite kit and of the MethylEdge Bisulfite Conversion System was the highest.

The inappropriate conversion of methylated cytosines to thymines should be as low as possible, otherwise, the methylation levels of the DNA can be underestimated. By using artificially methylated controls to generate percentage of methylation standard curves, we showed that the expected methylation levels of the controls were very close to the observed ones with Sanger sequencing (Fig 5). This is a demonstration of the ability of this method to accurately and consistently quantify CpG methylation. In order to analyze the chromatogram traces (.abi) we used ESME, an automated open-source Sanger methylation data analysis software package developed for the Human Epigenome [34]. This software only analyzes CpG dinucleotides and measures intensity by the area under the curve from both the thymine trace and the cytosine trace, calculating the percentage of cytosine methylation by the value of cytosine intensity divided by the total intensity [41].

Instead of direct sanger sequencing, cloning and sequencing of multiple colonies after bisulfite conversion is regarded as the reference method for the analysis of DNA methylation due to the richness of information provided. However, the quantitative resolution is limited by the numbers of clones analysed: when a small number of colonies is analysed, in the order of ten to twenty, it is difficult to obtain statistically meaningful results. In addition, this procedure is prone to a variety of biases distorting the quantitative results. Firstly, as in all PCR-based approaches there is a risk of biased amplification favouring usually completely methylated or unmethylated fragments. Secondly, the cloning procedure introduces another occasion for a potential selection bias [47]. On the other hand, the algorithms of ESME permit the quantitative analysis directly from four dye trace without the need for cloning, through alignment to a genomic reference sequence, trace off-set correction and normalization of signal intensities. In addition, although direct-BSP has low sensitivity, it provides more accurate detection of differences as low as 20% in methylation status in a single CpG [34].

The data generated from the Premium Bisulfite kit and the MethylEdge Bisulfite Conversion System showed higher correlation coefficients suggesting more accurate methylation quantitation. Therefore, these two kits were further used for bisulfite amplicon sequencing on an Illumina MiSeq platform. Fig 6 shows the observed methylation levels in correlation with the expected methylation levels. The λ-DNA control was able to fit linear lines with both the Promega and Diagenode kit with slightly higher corelation coefficient when using the Promega kit. Considering our results on the degradation effect, the conversion efficiency and conversion specificity, we selected the MethylEdge Bisulfite Conversion System (Promega) to be used for further analysis of our DMRs.

In 2009, our group published the first large-scale DMR identification study where more than 2000 regions of differential methylation between female whole blood and placental DNA were identified [23]. The discovery of biomarkers that can be used in the research of NIPT is very important since these are the tool to correctly discriminate between fetal and maternal DNA. It was speculated that these markers had the potential to be developed into targets for the NIPT of trisomy 21 and other aneuploidies. Indeed, in 2011, by using a subset of these DMRs on chromosome 21, our group was able to successfully detect trisomy 21 by applying MeDIP in combination with qPCR [48]. Two of the DMRs included in the diagnostic formula were analyzed for the first time at base resolution here, using NGS. We confirmed that EP6 and EP10 are both hypermethylated in the CVS and hypomethylated in the female whole blood (Figs 8 and 9). In agreement with this, EP6 was found from an independent study to be hypermethylated in the placenta compared to the blood regardless of the presence of disease or not [49].

In a recent study, it was shown that the MethPrimers (which are designed based on the idea that the non-CpG cytosines are unmethylated and thus converted) select negatively hypermethylated DNA sequences in the PCR step of the bisulfite assay, resulting in CpG methylation underestimation and non-CpG methylation masking [50]. The paper also concludes that the higher the methylation levels, the greater is the bias of the MethPrimers towards reducing the methylation levels. In our case, by using the MethPrimers we were able to prove that the CpGs of the two DMRs were hypermethylated in the CVS compared to the whole blood. Based on the conclusions of the aforementioned study, since the fetal DNA displays a higher non-CpG methylation we believe that our results (the methylation difference between the two tissues) would have been further enhanced if we had used methylation-insensitive primers (MIPs) with the CVS showing even higher methylation.

In this study, we compared the two sequencing methods, Sanger and NGS. We demonstrated that there is an increased accuracy of the methylation quantitation in the NGS approach which can be attributed to the digital quantitation of the data as opposed to the inaccuracies that can be introduced from the analog nature of the Sanger sequencing quantitation, as CpG methylation is a function of the area under the curve of the C and the T traces. In addition, the sequencing depth that was achieved with the NGS approach further contributed to robust quantitation and increased statistical power. The Illumina MiSeq is an attractive instrument for targeted bisulfite amplicon sequencing because of its short run times and low cost and also because its sensitive optics and base-calling algorithms allow for low-diversity samples to be successfully sequenced. In our case, MiSeq generated adequate data to sufficiently and accurately quantify DNA methylation.

A drop off in coverage was observed at the ends of our sequenced amplicons. This has been reported by other studies and is attributed to dephasing [51]. It was suggested that in bisulfite sequencing some regions have low bisulfite conversion, while other regions do not; some regions have low coverage, while other regions do not. It is unclear how these differences are related to DNA sequence structure (e.g., GC contents and repetitive regions) [52]. It is estimated that approximately 10% of the CpG sites in the genome will be hard to align after bisulfite conversion. Longer sequence reads are expected to align more accurately and increase the genome coverage [53]. As proposed by Masser et al., a sequencing depth of ≥1000 x is sufficient for accurate methylation quantitation in bisulfite amplicon sequencing [41]. In this study, the majority of the samples (>60%) have obtained more or close to 1000x coverage.

Our study includes a number of limitations: Even though the aim was to compare the four kits regarding their conversion efficiency, conversion specificity and degradation effect, other parameters have not been addressed, such as DNA purity and stability. Nevertheless, it was previously shown that potential impurities present after bisulfite reactions do not have any inhibitory effect in downstream PCR experiments [54]. Moreover, in the future, we plan to scale down the starting amount of DNA in order to reach the plasma quantities and eventually apply the procedure in plasma instead of whole blood. This will enable the testing of the bisulfite amplicon sequencing approach in NIPT by using DMRs between maternal and fetal DNA. In addition, the kits were compared based on the λ-DNA sequence as a reference control. In order to have a direct comparison of the results human DNA may have been a better choice to be used as reference. Finally, in order to draw conclusions about the interindividual variability of the methylation level of the DMRs, a large-scale study needs to be conducted.

## Conclusion

The kits studied in this project showed similar yet distinct results regarding DNA degradation, conversion efficiency and conversion specificity. For the purposes of our experiments, in our laboratoty and based on the results that are presented in this study, it was concluded that the best performance was observed with the MethylEdge Bisulfite Conversion System (Promega) followed by the Premium Bisulfite kit (Diagenode). Furthermore, two of our previously published DMRs were studied for the first time at single base resolution and their methylation difference between CVS and whole blood was confirmed. Finally, we have shown that bisulfite amplicon sequencing is a suitable approach for methylation analysis of specific regions.

## Supporting Information

S1 Table MiSeq run summaries. (DOCX)

S2 Table Number of reads from NGS. (DOCX)
