# Supplementary material for: Bisulfite Conversion of DNA: Performance Comparison of Different Kits and Methylation Quantitation of Epigenetic Biomarkers that Have the Potential to Be Used in Non-Invasive Prenatal Testing
Source: PLoS One. 2015 Aug 6;10(8):e0135058. doi: 10.1371/journal.pone.0135058 (PMC4527772; doi:10.1371/journal.pone.0135058)
Supplement: S1 Table — (DOCX) [file pone.0135058.s001.docx]

**Table S1. MiSeq run summaries**.

| Cluster Density (k/mm^2^) | Clusters PF (%) | Total paired reads PF (M) | Total % ≥Q30 |
| --- | --- | --- | --- |
| 680 ± 55 | 90.85 ± 21.71 | 15.19 | 96.6 |

Flow cell cluster densities, the percent of cluster passing filter (PF), total paired reads passing filter and total percent of reads above Q30.
