# Supplementary material for: Bisulfite Conversion of DNA: Performance Comparison of Different Kits and Methylation Quantitation of Epigenetic Biomarkers that Have the Potential to Be Used in Non-Invasive Prenatal Testing
Source: PLoS One. 2015 Aug 6;10(8):e0135058. doi: 10.1371/journal.pone.0135058 (PMC4527772; doi:10.1371/journal.pone.0135058)
Supplement: S2 Table — (DOCX) [file pone.0135058.s002.docx]

**Table S2. Number of reads from NGS.**

| Library | Kit | EP6 | EP10 | Lambda control 1 | Lambda control 2 |  | Library | Kit | EP6 | EP10 | Lambda control 1 | Lambda control 2 |
| --- | --- | --- | --- | --- | --- | --- | --- | --- | --- | --- | --- | --- |
| 1 | Diagenode | 65312 | 5285 | 12428 | 2912 |  | 49 | Epigentek | 75 | 7 | 119858 | 6 |
| 2 |  | 54016 | 4081 | 14898 | 280 |  | 50 |  | 16 | 0 | 61607 | 1 |
| 3 |  | 54619 | 4975 | 102484 | 298 |  | 51 |  | 55 | 3 | 53221 | 0 |
| 4 |  | 84133 | 6783 | 10741 | 372 |  | 52 |  | 80 | 6 | 53416 | 0 |
| 5 |  | 46653 | 5040 | 21161 | 322 |  | 53 |  | 43 | 99 | 58136 | 0 |
| 6 |  | 179 | 356 | 85961 | 4 |  | 54 |  | 49 | 19 | 56937 | 2 |
| 7 |  | 13120 | 972 | 29889 | 895 |  | 55 |  | 69 | 11 | 67339 | 2 |
| 8 |  | 20761 | 3814 | 29270 | 1951 |  | 56 |  | 6 | 0 | 51494 | 0 |
| 9 |  | 52365 | 4000 | 7618 | 1253 |  | 57 |  | 4 | 0 | 50765 | 0 |
| 10 |  | 78154 | 7266 | 18826 | 423 |  | 58 |  | 0 | 0 | 115 | 0 |
| 11 |  | 51955 | 6600 | 16272 | 520 |  | 59 |  | 61 | 3 | 63476 | 3 |
| 12 |  | 78568 | 8579 | 16476 | 443 |  | 60 |  | 19 | 1 | 37470 | 0 |
| 13 |  | 96995 | 8581 | 7183 | 468 |  | 61 |  | 666 | 796 | 59492 | 8 |
| 14 |  | 66370 | 3166 | 8365 | 372 |  | 62 |  | 1035 | 163 | 47520 | 14 |
| 15 |  | 89178 | 8680 | 41881 | 715 |  | 63 |  | 149 | 2 | 65329 | 2 |
| 16 |  | 45115 | 5444 | 18919 | 4213 |  | 64 |  | 22488 | 123 | 822 | 26 |
| 17 |  | 35533 | 3781 | 15860 | 1541 |  | 65 |  | 6 | 0 | 56446 | 0 |
| 18 |  | 35233 | 3066 | 18836 | 342 |  | 66 |  | 37 | 5 | 57722 | 2 |
| 19 |  | 61048 | 5806 | 18665 | 573 |  | 67 |  | 6 | 0 | 45852 | 2 |
| 20 |  | 74762 | 4079 | 16331 | 578 |  | 68 |  | 7 | 3 | 68568 | 0 |
| 21 |  | 55140 | 3299 | 9316 | 344 |  | 69 |  | 91 | 35 | 80424 | 0 |
| 22 |  | 55435 | 5956 | 17956 | 532 |  | 70 |  | 24937 | 113 | 2031 | 47 |
| 23 |  | 74168 | 2417 | 12368 | 509 |  | 71 |  | 62193 | 242 | 3476 | 281 |
| 24 |  | 49705 | 1708 | 14830 | 4010 |  | 72 |  | 49798 | 922 | 22128 | 1432 |
| 25 | Promega | 79445 | 13483 | 25586 | 998 |  | 73 | Qiagen | 10240 | 2010 | 60993 | 136 |
| 26 |  | 100530 | 6301 | 26247 | 1510 |  | 74 |  | 22531 | 2350 | 1779 | 84 |
| 27 |  | 90881 | 6870 | 19006 | 1338 |  | 75 |  | 20002 | 1128 | 1041 | 38 |
| 28 |  | 43578 | 3783 | 9716 | 483 |  | 76 |  | 9479 | 928 | 5462 | 27 |
| 29 |  | 55586 | 7958 | 10881 | 513 |  | 77 |  | 4822 | 1053 | 64 | 156 |
| 30 |  | 74159 | 7650 | 14700 | 1286 |  | 78 |  | 50856 | 11139 | 18325 | 924 |
| 31 |  | 52620 | 6916 | 11855 | 1147 |  | 79 |  | 2157 | 259 | 2851 | 249 |
| 32 |  | 36 | 20 | 26 | 0 |  | 80 |  | 14654 | 981 | 30895 | 455 |
| 33 |  | 62608 | 6131 | 20020 | 624 |  | 81 |  | 28 | 36 | 12170 | 24 |
| 34 |  | 50 | 2 | 11 | 4 |  | 82 |  | 10126 | 1578 | 5203 | 417 |
| 35 |  | 21 | 0 | 37951 | 0 |  | 83 |  | 10416 | 4100 | 11 | 532 |
| 36 |  | 69779 | 8367 | 12328 | 1307 |  | 84 |  | 3525 | 1316 | 2012 | 134 |
| 37 |  | 56009 | 13742 | 11625 | 852 |  | 85 |  | 2009 | 255 | 2184 | 113 |
| 38 |  | 45160 | 5252 | 6701 | 756 |  | 86 |  | 89 | 23 | 66 | 0 |
| 39 |  | 99788 | 19444 | 33990 | 2953 |  | 87 |  | 15604 | 1576 | 6124 | 545 |
| 40 |  | 59381 | 15755 | 41563 | 6332 |  | 88 |  | 57200 | 1328 | 36647 | 321 |
| 41 |  | 78700 | 4605 | 14394 | 2107 |  | 89 |  | 45397 | 6981 | 8108 | 304 |
| 42 |  | 74461 | 619 | 3219 | 394 |  | 90 |  | 12 | 3 | 60695 | 0 |
| 43 |  | 51267 | 3743 | 8121 | 1121 |  | 91 |  | 25491 | 6437 | 12932 | 212 |
| 44 |  | 67281 | 5817 | 10859 | 1678 |  | 92 |  | 48583 | 7135 | 12379 | 659 |
| 45 |  | 89577 | 5157 | 15667 | 1953 |  | 93 |  | 41737 | 9117 | 14810 | 435 |
| 46 |  | 59 | 0 | 73844 | 0 |  | 94 |  | 61824 | 13544 | 31347 | 393 |
| 47 |  | 115 | 36 | 90634 | 0 |  | 95 |  | 34 | 11 | 19931 | 2 |
| 48 |  | 20 | 3 | 121714 | 0 |  | 96 |  | 53184 | 4351 | 24249 | 897 |

The number of reads that was obtained for each sample is shown for each kit. The samples that had very low concentration were not processed further for library preparation and therefore gave no reads.
